# Development and Evaluation of a Novel ^99m^Tc-Labeled Annexin A5 for Early Detection of Response to Chemotherapy

**DOI:** 10.1371/journal.pone.0081191

**Published:** 2013-12-04

**Authors:** Kazuma Ogawa, Katsuichi Ohtsuki, Tomomi Shibata, Miho Aoki, Morio Nakayama, Yoji Kitamura, Masahiro Ono, Masashi Ueda, Tomoki Doue, Masahisa Onoguchi, Kazuhiro Shiba, Akira Odani

**Affiliations:** 1 Graduate School of Medical Sciences, Kanazawa University, Kanazawa, Japan; 2 Department of Cardiovascular Medicine, Kameoka Hospital, Kameoka, Japan; 3 Graduate School of Biomedical Sciences, Nagasaki University, Nagasaki, Japan; 4 Advanced Science Research Center, Kanazawa University, Kanazawa, Japan; 5 Graduate School of Pharmaceutical Sciences, Kyoto University, Kyoto, Japan; 6 Graduate School of Medicine, Dentistry, and Pharmaceutical Sciences, Okayama University, Okayama, Japan; 7 Department of Cardiology, Rinku General Medical Center, Izumisano, Japan; Centro Nacional de Investigaciones Oncológicas (CNIO), Spain

## Abstract

^99m^Tc-HYNIC-annexin A5 can be considered as a benchmark in the field of apoptosis imaging. However, ^99m^Tc-HYNIC-annexin A5 has characteristics of high uptake and long retention in non-target tissues such as kidney and liver. To minimize this problem, we developed a novel ^99m^Tc-labeled annexin A5 using a bis(hydroxamamide) derivative [C_3_(BHam)_2_] as a bifunctional chelating agent, and evaluated its usefulness as an imaging agent for detecting apoptosis. The amino group of C_3_(BHam)_2_ was converted to a maleimide group, and was coupled to thiol groups of annexin A5 pretreated with 2-iminothiolane. ^99m^Tc labeling was performed by a ligand exchange reaction with ^99m^Tc-glucoheptonate. Biodistribution experiments for both ^99m^Tc-C_3_(BHam)_2_-annexin A5 and ^99m^Tc-HYNIC-annexin A5 were performed in normal mice. In addition, in tumor-bearing mice, the relationship between the therapeutic effects of chemotherapy (5-FU) and the tumor accumulation of ^99m^Tc-C_3_(BHam)_2_-annexin A5 just after the first treatment of 5-FU was evaluated. ^99m^Tc-C_3_(BHam)_2_-annexin A5 was prepared with a radiochemical purity of over 95%. In biodistribution experiments, ^99m^Tc-C_3_(BHam)_2_-annexin A5 had a much lower kidney accumulation of radioactivity than ^99m^Tc-HYNIC-annexin A5. In the organs for metabolism, such as liver and kidney, radioactivity after the injection of ^99m^Tc-HYNIC-annexin A5 was residual for a long time. On the other hand, radioactivity after the injection of^ 99m^Tc-C_3_(BHam)_2_-annexin A5 gradually decreased. In therapeutic experiments, tumor growth in the mice treated with 5-FU was significantly inhibited. Accumulation of ^99m^Tc-C_3_(BHam)_2_-annexin A5 in tumors significantly increased after 5-FU treatment. The accumulation of radioactivity in tumor correlated positively with the counts of TUNEL-positive cells. These findings suggest that ^99m^Tc-C_3_(BHam)_2_-annexin A5 may contribute to the efficient detection of apoptotic tumor response after chemotherapy.

## Introduction

Annexin A5 is a 36-kDa human protein with a high affinity for phosphatidyl serine (PS). PS is normally retained on the intracellular face of the cell membrane. However, when cells undergo apoptosis (cell death), this distribution is altered, that is, PS is rapidly exposed to the outside of the cell membrane. For that reason, radiolabeled annexin A5 can be used to detect cell death *in vivo* [1], [2].

^99m^Tc is an ideal radionuclide for scintigraphic imaging applications due to its excellent physical properties, low cost, and ready availability because of generator-produced nuclide. Since most polypeptides do not possess binding sites to form ^99m^Tc chelates of high *in vivo* stability, appropriate chelating molecules are incorporated into polypeptide molecules to prepare ^99m^Tc-labeled peptides for *in vivo* applications. Hydrazinonicotinamide (HYNIC) is one of the most attractive bifunctional chelating agents for the labeling of peptides and proteins with ^99m^Tc. It was reported that HYNIC acts as a monodentate or bidentate ligand to form a mixed ligand ^99m^Tc complex in the presence of appropriate coligands [3]. Several coligands have been reported such as glucoheptonate, tricine, ethylene diamine diacetic acid (EDDA), and ternary ligand systems containing tricine and water-soluble phosphines or tricine and imine-N-containing heterocycles. Tricine has been used most widely for protein labeling, since it provides ^99m^Tc-HYNIC-labeled polypeptides with high radiochemical yields and high specific activities in a short reaction time [4], [5]. ^99m^Tc-HYNIC-annexin A5 can be considered as a benchmark in the field of apoptosis imaging since this tracer is the most extensively investigated and best characterized apoptosis-detecting radioligand to date [6]. Although ^99m^Tc-HYNIC-annexin A5 has been successfully used in many studies, it has characteristics of high uptake and long retention in non-target tissues such as kidney and liver [7].

On the other hand, bis(hydroxamamide) derivative, *N,N’*-trimethylenedibenzohydroxamamide ligand [C_3_(BHam)_2_] can also form a stable ^99m^Tc complex over a wide pH range under mild reaction conditions within short reaction times [8]. The complexation yield is high (over 95%) at ligand concentrations as low as 2.5×10^−6^ M. In a previous study, when ^99m^Tc-C_3_(BHam)_2_-IgG was administered to mice, the radioactivity in liver was not residual [9]. This result suggests that the radiometabolite of ^99m^Tc-C_3_(BHam)_2_-IgG could be eliminated rapidly from metabolic organs.

The aim of this study is to develop and evaluate a novel ^99m^Tc-labeled annexin A5, which shows lower the radioactivity levels in non-target tissue, such as kidney and liver. Then, we assumed that the clearance of radiometabolites in these organs might play a crucial role, and hypothesized that the radioactivity after administration of ^99m^Tc-C_3_(BHam)_2_-conjugated annexin A5, ^99m^Tc-C_3_(BHam)_2_-annexin A5, could be rapidly eliminated from kidney and liver. ^99m^Tc-C_3_(BHam)_2_-annexin A5 was prepared and its bioactivity and biodistribution was compared to that of ^99m^Tc-HYNIC-annexin A5. In addition, to evaluate whether ^99m^Tc-C_3_(BHam)_2_-annexin A5 is able to detect apoptosis as an early response of chemotherapy, the relationship between the therapeutic effects of chemotherapy and the tumor accumulation of ^99m^Tc-C_3_(BHam)_2_-annexin A5 after injection of 5-FU in tumor-bearing mice, and the correlation between tumor accumulation or intratumoral distribution of ^99m^Tc-C_3_(BHam)_2_-annexin A5 and the number of TUNEL-staining positive cells were evaluated.

## Materials and Methods

### Materials

Thin layer chromatography (TLC) analyses were performed with silica plates (Silica gel 60, Merck KGaA, Darmstadt, Germany) with saline as a developing solvent. Size-exclusion (SE)-HPLC analyses were performed with a TSK-GEL Super SW3000 column (4.6×300 mm, TOSOH, Tokyo, Japan) at a flow rate of 0.3 mL/min with 0.1 M phosphate buffer (pH 6.8). [^99m^Tc]Pertechnetate (^99m^TcO_4_^-^) was eluted in saline solution from generators (Nihon Medi-Physics Co., Ltd., Tokyo, Japan). Annexin A5 was generously provided by Kowa Co., Ltd. (Nagoya, Japan). Other reagents were of reagent grade and used as received.

### Preparation of C_3_(BHam)_2_-annexin A5

**Figure 1:**
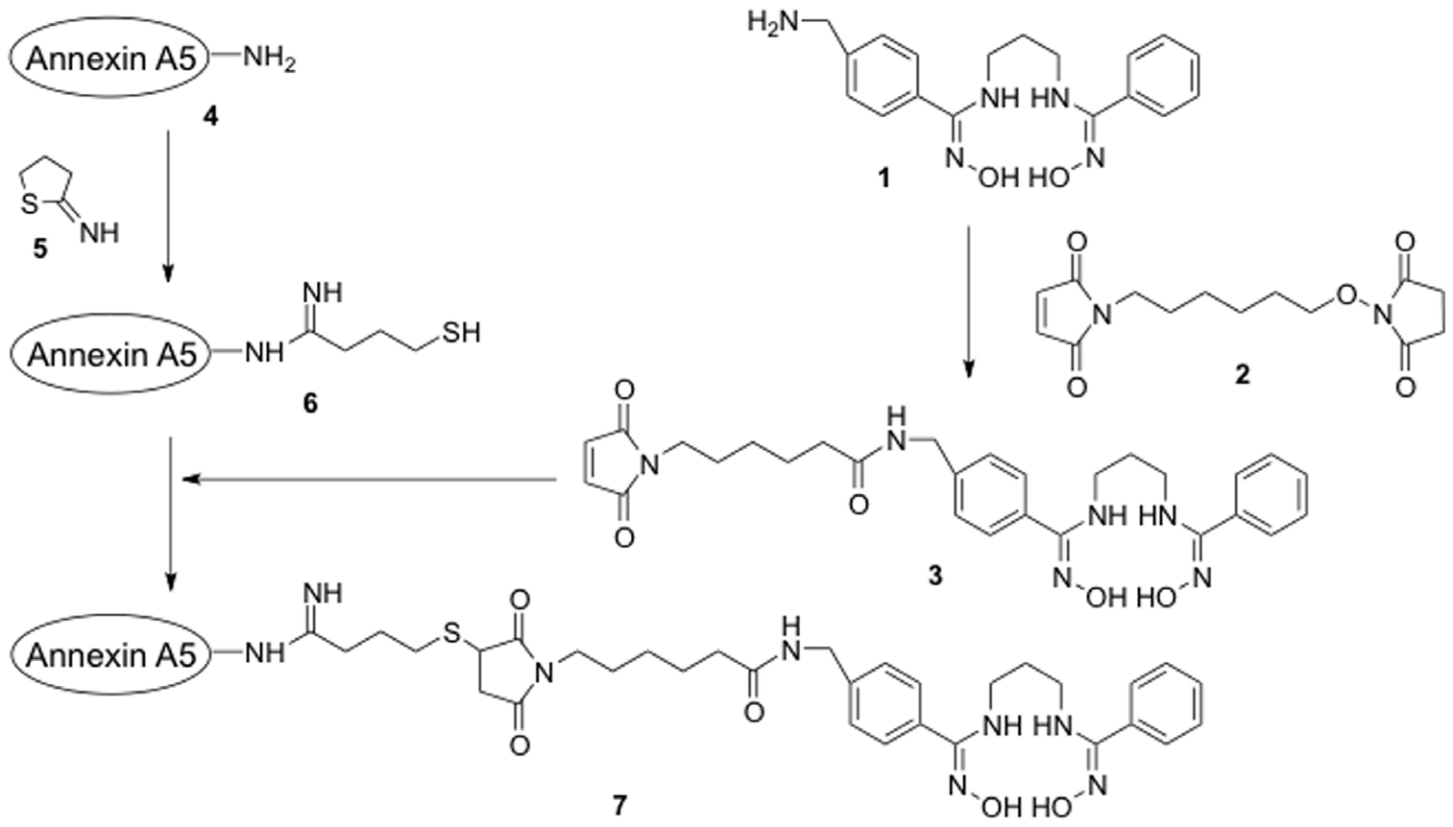
Preparation of C_3_(BHam)_2_-annexin A5.

C_3_(BHam)_2_-Annexin A5 (**7**) was prepared according to the procedure outlined in Figure 1.

4′-Aminomethyl-*N,N′*-trimethylenedibenzohydroxamamide [NH_2_-C_3_(BHam)_2_] (**1**) was prepared according to a previously reported procedure [9]. Compound **1** (1 mg) was dissolved in 36 µL of *N*,*N*-dimethylformamide (DMF), and *N*-(6-maleimidocaproyloxy)succinimide (EMCS) (**2**), dissolved in 36 µL of DMF, was added to the solution of compound **1**. Annexin A5 (2 mg) was dissolved in 800 µL of 0.16 M borate buffer containing 2 mM EDTA (pH 8.0). 2-Iminothiolane (2-IT) (**5**) (80 µg) dissolved in 40 µL of 0.16 M borate buffer containing 2 mM EDTA (pH 8.0) was added to the annexin A5 solution. After being stirred for 1 h at room temperature, the reaction mixture was purified by PD-10 column (GE Healthcare UK Ltd., Buckinghamshire, England). The fraction (1.5 mL) containing 2-iminothiolane conjugated annexin A5 (**6**) was determined by SE-HPLC monitoring of UV adsorption at a wavelength of 280 nm, and collected. An aqueous solution of 1 M NaH_2_PO_4_ (192 µL) and 16 µL of the reaction mixture (**3**) described above was added to the compound **6** solution (1.5 mL), and the reaction mixture was stirred for 2 h at room temperature. After addition of 100 µL of iodoacetamide (10 mg/mL) to alkylate the unreacted thiol groups, the solution was further gently agitated for 10 min. The C_3_(BHam)_2_-annexin A5 (**7**) conjugate was separated from unreacted small molecules by PD-10 column. The number of C_3_(BHam)_2_ ligands introduced per molecule of annexin A5 was estimated by measuring the number of thiol groups in the annexin A5 before and after the conjugation reaction with compound **3** using 4,4′-dithiodipyridine [10].

### ^99m^Tc Labeling of C_3_(BHam)_2_-annexin A5

C_3_(BHam)_2_-Annexin A5 was labeled with ^99m^Tc by a ligand-exchange reaction with ^99m^Tc-glucoheptonate.

^99m^TcO_4_^-^ (100L, 37-740 MBq) was added to 0.2 mg of lyophilized Sn(II) glucoheptonate, which was prepared by the method described previously by Yamamura et al [11], and allowed to stand for 15 min at room temperature. Formation of ^99m^Tc-glucoheptonate was ascertained by TLC developed with acetone. The ^99m^Tc-glucoheptonate solution was added to the C_3_(BHam)_2_-annexin A5 solution (334–1407 µg/mL) and the mixture was incubated for 1 h at room temperature. The radiochemical purities of ^99m^Tc-C_3_(BHam)_2_-annexin A5 were determined by TLC and SE-HPLC. When the radiochemical purity was less than 95%, the reaction mixture was purified with a PD-10 column with saline as the eluate.

### Cell Binding Assay

The bioactivity of ^99m^Tc-labeled annexin A5 was determined by their binding to erythrocytes, according to a previously reported procedure [12]. In brief, ^99m^Tc-C_3_(BHam)_2_-annexin A5 or ^99m^Tc-HYNIC-annexin A5, which was prepared according the previously reported procedure [7], at 10 nmol/L final concentration was added to duplicated tubes containing a final volume of 1 mL of buffer HNKGB (10 mM HEPES-Na, pH 7.4, 136 mM NaCl, 2.7 mM KCl, 5 mM glucose, and 1 mg/mL BSA) plus 2.5 mmol/L CaCl_2_. One tube then received 4.2×10^8^ erythrocytes in 100 µL. The other tube then received an equal volume of buffer, and samples were incubated for 15 min at room temperature. After centrifugation at 8,320 *g* for 3 min, the radioactivity of the supernatant was measured with an auto well gamma counter (ARC-380; Aloka, Tokyo, Japan). The binding ratios were determined as follows: Radioactivity bound to erythrocytes (%) = (1 – [radioactivity of supernatant in the presence of erythrocytes]/[radioactivity of supernatant in absence of erythrocytes]) ×100.

### Biodistribution Experiments

The animal experimental protocols used were approved by the Committee on Animal Experimentation of Kanazawa University (Permit Number: AP-132633). Experiments with animals were conducted in strict accordance with the Guidelines for the Care and Use of Laboratory Animals of Kanazawa University. The animals were housed with free access to food and water at 23°C with a 12-hour alternating light/dark schedule. Biodistribution experiments were performed by intravenous administration of 100 µL of ^99m^Tc-labeled Annexin A5 (37 kBq, 1.5 µg Annexin A5) into 6-week-old male ddY mice (28–30 g, Japan SLC, Inc., Hamamatsu, Japan). Groups of five mice each were sacrificed by decapitation at 10 min, 1, 3, and 6 h after injection. Organs of interest were removed and weighed and the radioactivity counts were determined with an auto well gamma counter and corrected for background radiation and physical decay during counting.

### Preparation of Tumor-Bearing Mice and Chemotherapy

Colon-26, a colorectal adenocarcinoma cell line derived from BALB/c mice, was used (TKG 0518, Cell Resource Center for Biomedical Research, Tohoku University, Sendai, Japan) [13]. To produce tumors, approximately 3×10^6^ of the prepared colon-26 cells in HEPES-buffered saline solution with 1% methylcellulose were injected subcutaneously into the dorsum of 6-week-old female BALB/c mice (15–20 g, Japan SLC, Inc.).

The mice were randomly distributed in the experimental groups. At 6 and 13 days after inoculation of the tumor cells, 5-fluorouracil (5-FU, Nacalai Tesque, Kyoto, Japan) was injected intraperitoneally at a dose of 100 mg/kg or 150 mg/kg. A group of saline-injected mice served as an untreated control group. Tumor size was measured with a slide caliper in two dimensions three times per week. Individual tumor volumes (V) were calculated by the formula V =  [length×(width)^2^×3.14]/6 and compared with the values on the day of treatment (relative tumor volume).

### Evaluation of Tumor Uptake of ^99m^Tc-C_3_(BHam)_2_-annexin A5 and ^99m^Tc-HYNIC-annexin A5

At 6 days after inoculation of the colon-26 tumor cells into the mice, 5-FU was injected intraperitoneally at a dose of 100 mg/kg or 150 mg/kg. A group of saline-injected mice served as a control group. At 24 h after treatment of 5-FU, ^99m^Tc-C_3_(BHam)_2_-annexin A5 (37 kBq, 1.5 µg annexin A5) was administered intravenously into the tumor-bearing mice. In the case of ^99m^Tc-HYNIC-annexin A5, a 150 mg/kg of 5-FU treated group and a control group were used. The mice were sacrificed by decapitation at 4 h after injection of ^99m^Tc-C_3_(BHam)_2_-annexin A5 or ^99m^Tc-HYNIC-annexin A5. The tumors were removed and weighed and the radioactivity counts were determined with an auto well gamma counter. After the counts were determined, the tumors were embedded in Tissue-Tek® O.C.T. compound medium (Sakura Finetek USA, Inc., Torrance, CA, USA) and frozen. Tumor sections 8 µm in size were obtained using a cryostat (HM 525 Cryostat, Thermo Fisher Scientific Inc., Waltham, MA, USA) for histological analysis.

### TUNEL Staining

Tumor sections were stained with terminal deoxynucleotidyl transferase-mediated deoxyuridine triphosphate nick-end labeling (TUNEL) using a commercially available kit (*In situ* Apoptosis Detection Kit, Takara Bio Inc., Otsu, Japan) according to the manufacturer's protocol. TUNEL-stained tissue sections were observed under a fluorescence microscope (BZ-9000, Keyence, Osaka, Japan). The number of TUNEL-positive cells was counted on 10 randomly chosen fields for each section.

### *Ex Vivo* Autoradiographic Experiments

^99m^Tc-C_3_(BHam)_2_-annexin A5 (11.1 MBq) was intravenously administered to 5-FU (150 mg/kg) treated tumor bearing mice or non-treatment tumor bearing mice as a control group, which were prepared by the above-mentioned method. At 4 hours after injection of ^99m^Tc-C_3_(BHam)_2_-annexin A5, the tumors were removed, embedded in Tissue-Tek® O.C.T. compound medium, and frozen. Serial frozen sections 8 µm in size were obtained using a cryostat. The sections were exposed for 12 hours on an imaging plate (BAS-SR, Fujifilm, Tokyo, Japan), and the radioactivity of each section was determined using a bio-imaging analyzer (BAS5000, Fujifilm). Another set of tissue sections adjacent to those used for autoradiography was stained with TUNEL.

### Statistical evaluation

A one-way analysis of variance (ANOVA) followed by Dunnett's post hoc test compared with the untreated control group was used for the experiments of 5-FU chemotherapy and evaluation of tumor uptake of ^99m^Tc-C_3_(BHam)_2_-Annexin A5. Tumor uptake between control group and 5-FU treated group after injection of ^99m^Tc-C_3_(BHam)_2_-Annexin A5 was compared using Students' *t* test. Results of *p*<0.05 were considered statistically significant.

## Results

### Preparation of ^99m^Tc-C_3_(BHam)_2_-annexin A5

C_3_(BHam)_2_-annexin A5 conjugate was prepared by converting the amine group of NH_2_-C_3_(BHam)_2_ (**1**) into a maleimide group with EMCS (**2**), followed by a conjugation reaction of the maleimide group with the thiol group of Annexin A5 with 2-IT. By measuring the thiol groups, the number of C_3_(BHam)_2_ ligands introduced per molecule of annexin A5 was estimated to be 0.7. ^99m^Tc-C_3_(BHam)_2_-annexin A5 was prepared by a ligand exchange reaction from ^99m^Tc-glucoheptonate with radiochemical yields of 47-97% and radiochemical purity of over 95%.

### Cell Binding Assay

In a cell binding assay to determine the bioactivity of ^99m^Tc-labeled annexin A5, the percentages of ^99m^Tc-C_3_(BHam)_2_-annexin A5 and ^99m^Tc-HYNIC-annexin A5 bound to erythrocytes were 78.92±0.63 and 81.35±0.89, respectively. These values of both types of ^99m^Tc-labeled annexin A5 indicated that their bioactivities are comparable.

### Biodistribution Studies

Table 1 lists the biodistribution of ^99m^Tc-C_3_(BHam)_2_-annexin A5 and ^99m^Tc-HYNIC-annexin A5 in normal mice. ^99m^Tc-C_3_(BHam)_2_-annexin A5 had remarkably lower kidney accumulation of radioactivity than ^99m^Tc-HYNIC-annexin A5. In the organs for metabolism, such as liver and kidney, the radioactivity of ^99m^Tc-HYNIC-annexin A5 was residual for a long time. On the other hand, radioactivity in these tissues after ^99m^Tc-C_3_(BHam)_2_-annexin A5 administration gradually decreased. However, the blood clearance of ^99m^Tc-C_3_(BHam)_2_-annexin A5 was slower when compared with that of ^99m^Tc-HYNIC-annexin A5. ^99m^Tc-C_3_(BHam)_2_-annexin A5 showed a higher initial uptake in liver than ^99m^Tc-HYNIC-annexin A5.

**Table 1:** Comparative biodistribution of radioactivity at 10^99m^Tc-C_3_(BHam)_2_-annexin A5 and ^99m^Tc-HYNIC-annexin A5 in mice.

Tissue	Time after injection			
	10 min	1 h	3 h	6 h
^99m^Tc-C_3_(BHam)_2_-annexin A5				
Blood	21.12 (1.86)	6.75 (0.45)	4.37 (0.38)	2.75 (0.33)
Liver	10.22 (1.33)	10.30 (1.82)	7.53 (0.56)	4.99 (0.61)
Kidney	34.84 (2.58)	32.14 (2.35)	16.05 (1.60)	10.25 (2.03)
Intestine	1.31 (0.07)	2.94 (0.53)	7.23 (0.69)	10.56 (0.62)
Spleen	9.07 (1.25)	13.49 (2.29)	10.76 (1.81)	5.58 (1.92)
Pancreas	1.16 (0.12)	1.22 (0.13)	1.07 (0.19)	0.65 (0.08)
Lung	13.19 (3.44)	7.88 (1.25)	5.08 (0.82)	2.83 (0.53)
Heart	6.64 (2.89)	4.16 (0.57)	2.94 (0.34)	1.64 (0.26)
Stomach†	0.72 (0.05)	1.04 (0.20)	1.15 (0.31)	0.81 (0.16)
^99m^Tc-HYNIC-annexin A5				
Blood	3.39 (0.34)	0.51 (0.05)	0.29 (0.02)	0.19 (0.02)
Liver	4.58 (0.35)	6.01 (0.54)	5.99 (0.46)	6.21 (0.68)
Kidney	104.98 (12.47)	114.18 (13.32)	113.52 (16.39)	108.52 (12.83)
Intestine	0.85 (0.17)	0.77 (0.09)	1.10 (0.32)	1.56 (0.05)
Spleen	6.20 (0.75)	7.15 (0.96)	7.32 (0.42)	6.82 (2.12)
Pancreas	0.93 (0.11)	0.71 (0.26)	0.55 (0.09)	0.55 (0.07)
Lung	4.77 (0.26)	2.37 (0.41)	1.96 (0.36)	1.60 (0.53)
Heart	2.05 (0.32)	1.21 (0.17)	1.04 (0.07)	1.00 (0.19)
Stomach†	0.37 (0.06)	0.45 (0.05)	0.49 (0.12)	0.57 (0.05)

Data are expressed as % injected dose per gram tissue. Each value represents the mean (SD) of 4-5 mice.

† Data are expressed as % injected dose.

### Tumor Accumulation of ^99m^Tc-C_3_(BHam)_2_-annexin A5 and ^99m^Tc-HYNIC-annexin A5 after Chemotherapy

**Figure 2:**
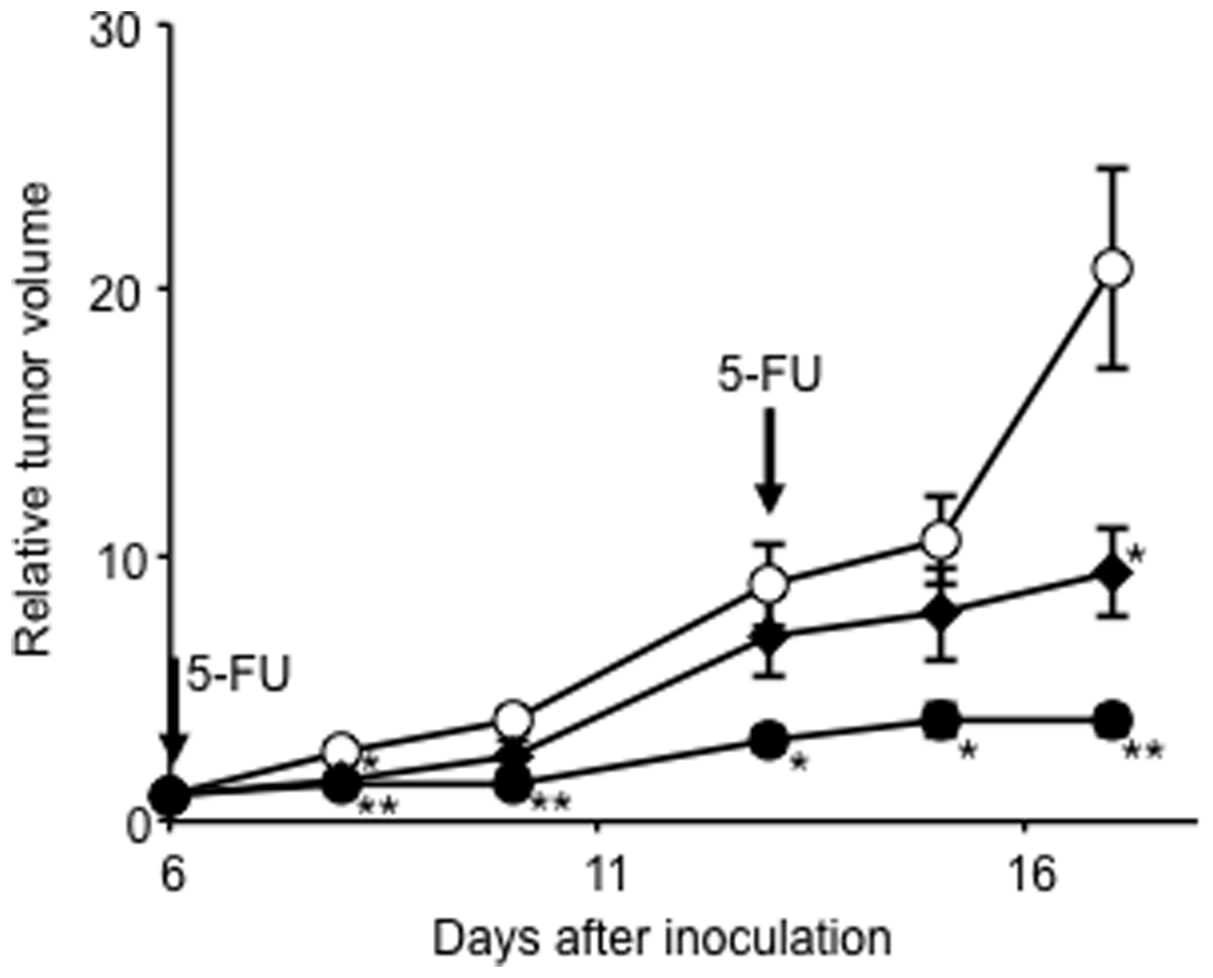
Tumor grouth curves. Curves depicting inhibition of the growth of colon-26 in treatment with 150 mg/kg of 5-FU (closed circles) or 100 mg/kg of 5-FU (closed diamonds) compared with untreated control group (open circles). Data are expressed as tumor volume relative to that on the day of treatment (mean ± SEM for 5 mice). Significance was determined using one-way ANOVA followed by Dunnett's post hoc test (**p<0.01, *p<0.05 vs. control group).

**Figure 3:**
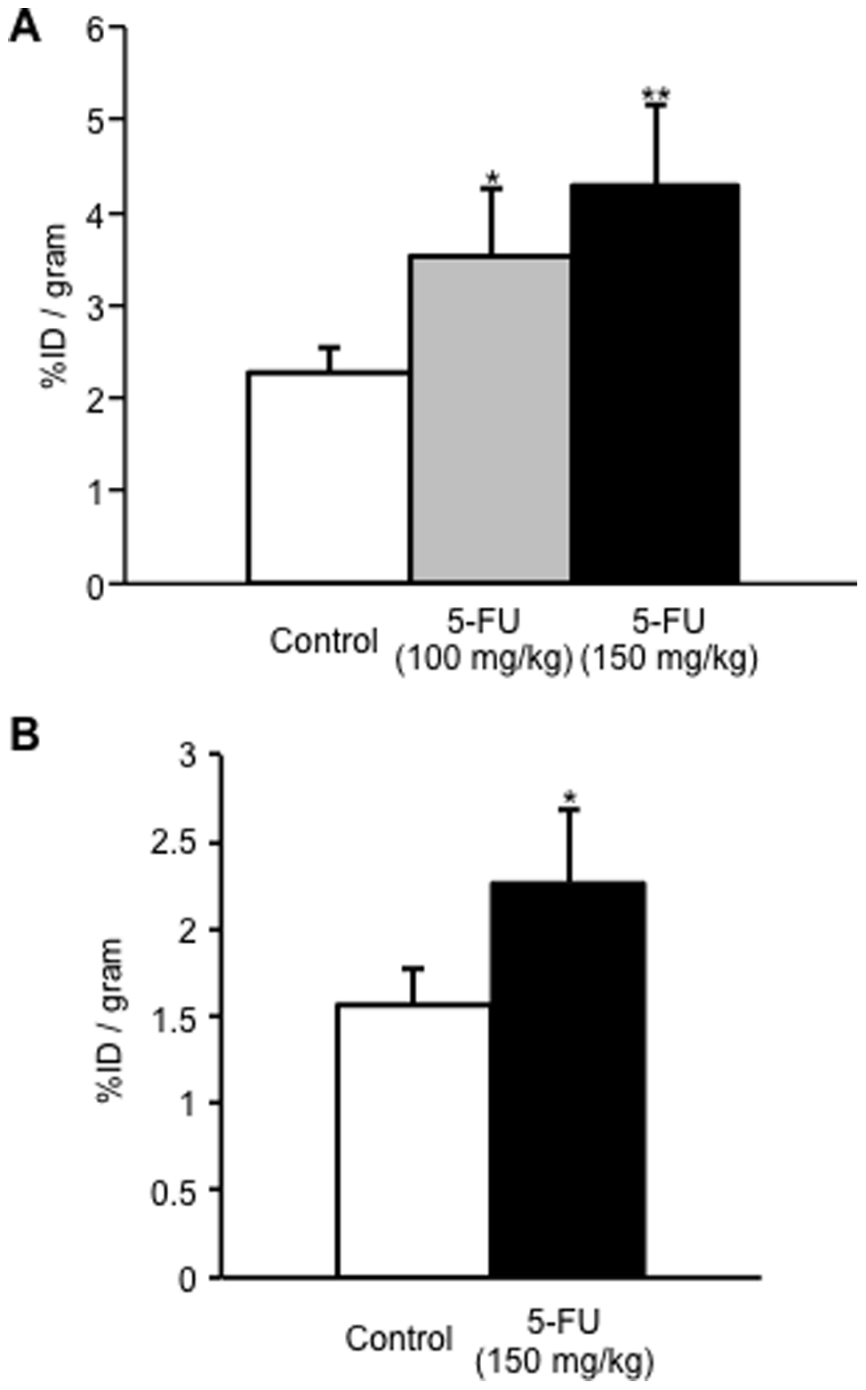
Tumore uptake. Comparison of tumor uptake of (A) ^99m^Tc-C_3_(BHam)_2_-annexin A5 (mean ± SD for 4-6 mice) and (B) ^99m^Tc-HYNIC-annexin A5 (mean ± SD for 6 mice) at 4 h after injection after 5-FU treatment or non-treatment. In the case of ^99m^Tc-C_3_(BHam)_2_-annexin A5, significance was determined using one-way ANOVA followed by Dunnett's post hoc test (**p<0.01, *p<0.05 vs. control group). In the case of ^99m^Tc-HYNIC-annexin A5, significance was determined using Students' t test (*p<0.05 vs. control group).

**Figure 4:**
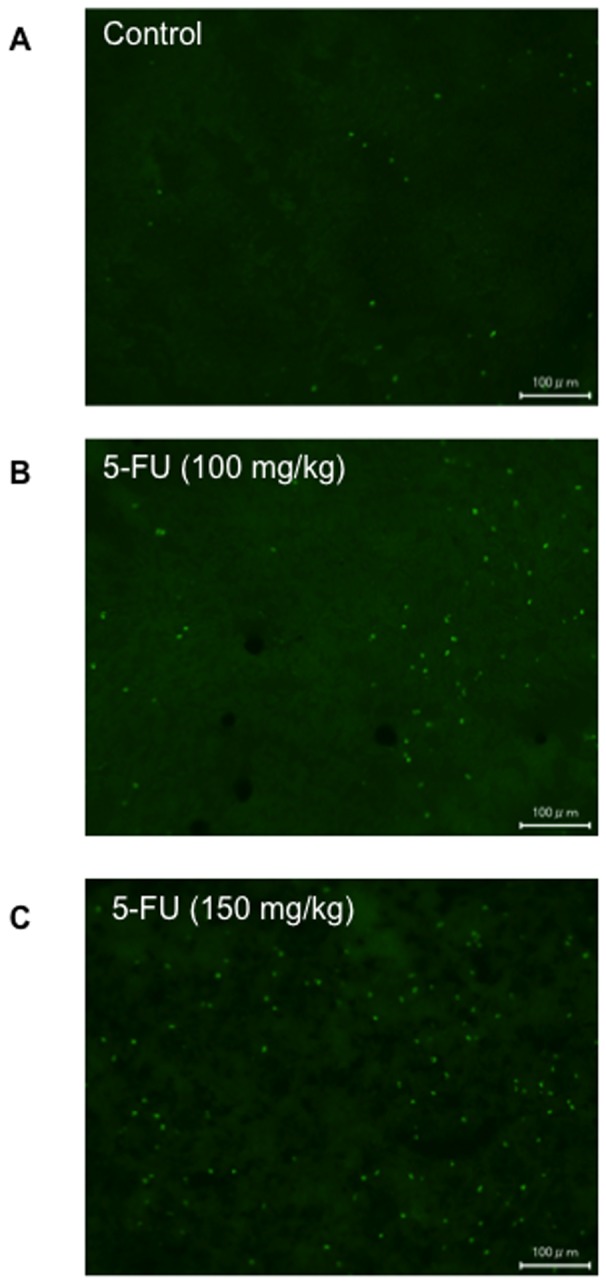
TUNEL-stained images. Representative TUNEL-stained images of tumor specimen in control mouse (A), 100 mg/kg of 5-FU-treated mouse (B), and 150 mg/kg of 5-FU-treated mouse (C). Scale bar  = 100 µm.

**Figure 5:**
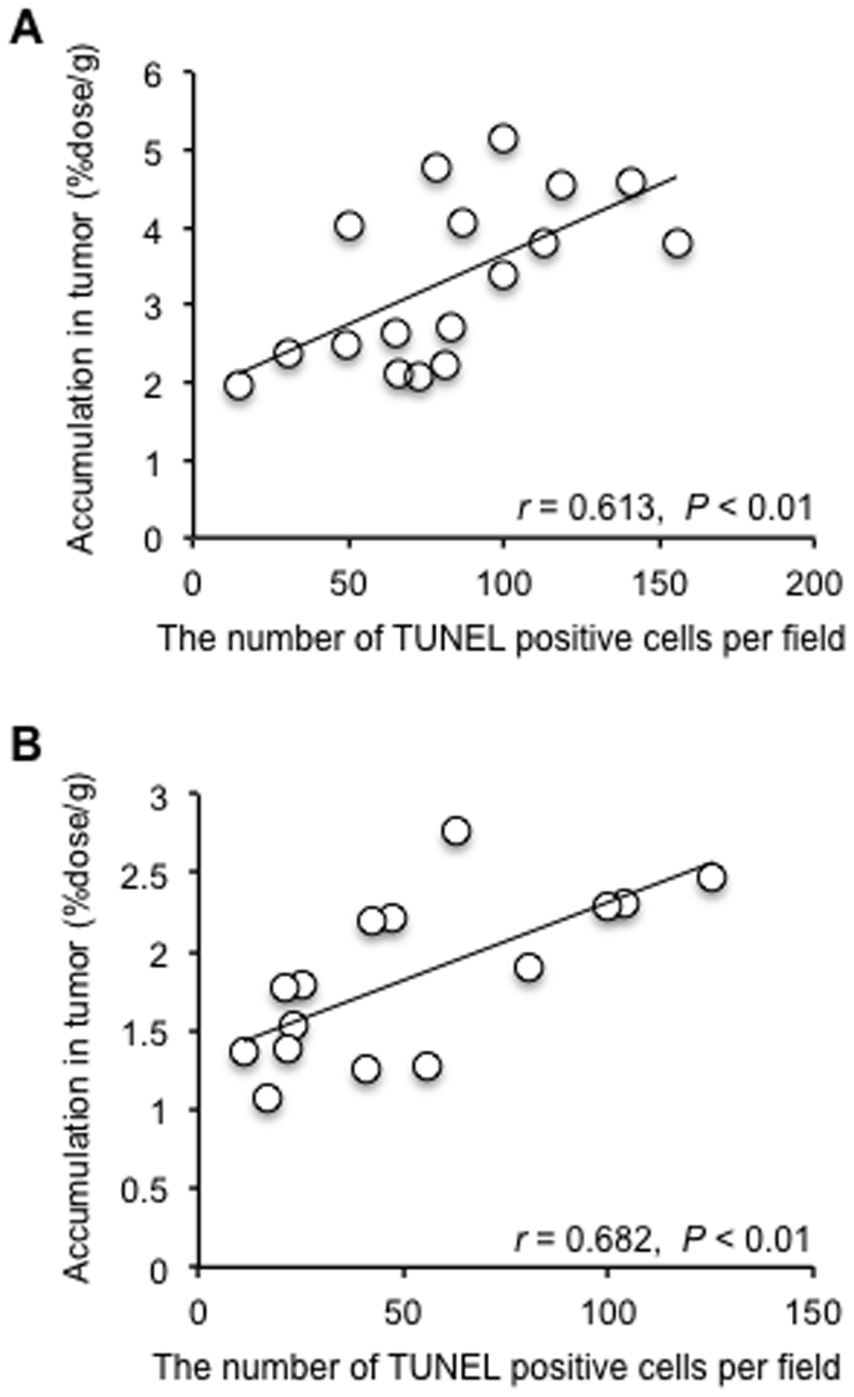
Correlation between TUNEL-positive cells and tumor uptake. Correlation between the number of TUNEL-positive cells in tumor after first 5-FU therapy and ^99m^Tc-C_3_(BHam)_2_-annexin A5 accumulation (%dose/g) (A) or ^99m^Tc-HYNIC-annexin A5 accumulation (%dose/g) (B) in corresponding tumor tissue.

Tumor volume as a function of time is shown in Figure 2. The colon-26 tumor cells proliferated exponentially in the untreated control group. In mice treated with 5-FU, especially the 150 mg/kg dose injected group, tumor growth was significantly inhibited compared to that of the untreated group. When ^99m^Tc-C_3_(BHam)_2_-annexin A5 was administered into tumor-bearing mice after 24 h of the first 5-FU treatment (at 7 days after inoculation), the accumulation of ^99m^Tc-C_3_(BHam)_2_-annexin A5 in tumor tissue was significantly higher than that of the untreated group (Figure 3A). The accumulation of ^99m^Tc-HYNIC-annexin A5 in tumor tissue was also significantly higher than in that of the untreated group (Figure 3B). At the same time, the number of TUNEL-positive cells in the tumor sections of the 5-FU-treated group was greater than that of the untreated control group (Figure 4). In both the case of ^99m^Tc-HYNIC-annexin A5 and that of ^99m^Tc-C_3_(BHam)_2_-annexin A5, the values of percent injected dose/gram in the tumor correlated well with the counts of TUNEL-staining positive cells in corresponding tissue sections (Figure 5).

### *Ex Vivo* Autoradiographic Experiments

**Figure 6:**
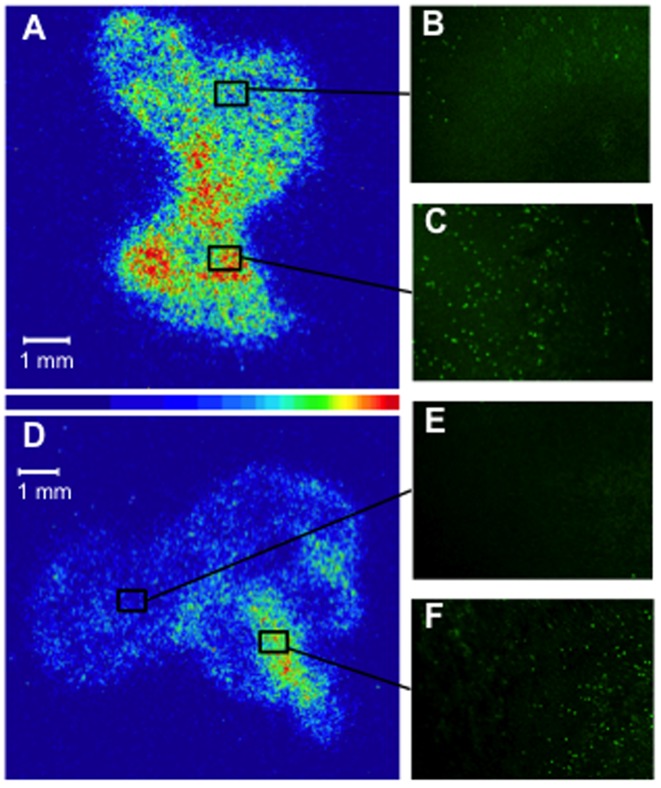
Autoradiography. Representative autoradiographic images (A, D) and TUNEL-staining images (B, C, E, F) for adjacent tumor sections from mice treated with 5-FU (A, B, C) or non-treatment mice (D, E, F). Scale bar  = 1 mm.

**Figure 7:**
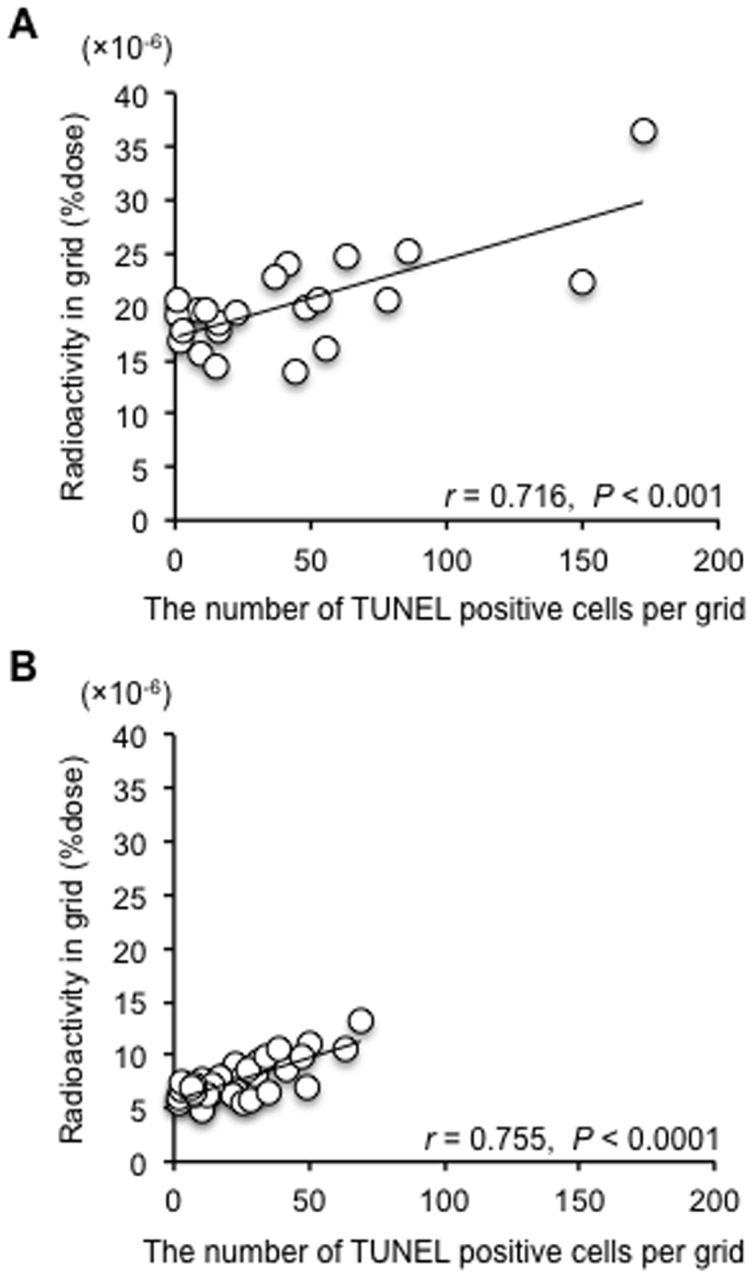
Correlation between TUNEL-positive cells and radioacitvity in tumor section. Correlation between the number of TUNEL-positive cells in each grid (0.45 mm×0.55 mm) of a tumoral section and ^99m^Tc-C_3_(BHam)_2_-annexin A5 accumulation (%dose) determined by autoradiography in each corresponding grid of an adjacent section from mice treated with 5-FU (A) or non-treatment mice (B).

Autoradiographic images of tumoral sections at 4 hours after injection of ^99m^Tc-C_3_(BHam)_2_-annexin A5 and corresponding TUNEL-staining images in adjacent sections are shown in Figure 6. These images show that the accumulation of radioactivity in tumors of 5-FU treated mice was higher compared to that in non-treatment control mice. Moreover, the sites of higher accumulation of radioactivity and the sites of intense positive TUNEL staining seem to match. To investigate whether the intratumoral localization of ^99m^Tc-C_3_(BHam)_2_-annexin A5 correlated with the sites of drug-induced apoptosis cells, we divided the autoradiographic and TUNEL-staining images of tumor sections using grids (grid size; 0.45 mm×0.55 mm) and determined the radioactivity and number of TUNEL-positive cells in each grid. The results showed that the intratumoral accumulations of radioactivity correlated well with the counts of TUNEL-staining positive cells in corresponding grids (Figure 7).

## Discussion

Tetradentate ligands, such as N_3_S and N_2_S_2_ coordination molecules, form stable ^99m^Tc complexes with the [Tc = O]^3+^ core. In the field of apoptosis imaging, a ^99m^Tc complex with N_2_S_2_ ligand-conjugated annexin A5 had been developed and evaluated in a previous paper [14], [15]. However, most of these ligands could require harsh conditions such as high pH or high temperature to prepare ^99m^Tc complexes with high radiochemical yields. Thus, ^99m^Tc labeling of annexin A5 with a N_2_S_2_ ligand was performed using the preformed chelate approach. This method requires multiple steps and purification, resulting in a 25-30% overall radiochemical yield [6]. In this study, we developed a new ^99m^Tc-labeled annexin A5, ^99m^Tc-C_3_(BHam)_2_-annexin A5, using a C_3_(BHam)_2_ ligand, which can form a ^99m^Tc complex over a wide pH range under mild reaction conditions. Actually, the C_3_(BHam)_2_ ligand was introduced into annexin A5 before radiolabeling, and ^99m^Tc-C_3_(BHam)_2_-annexin A5 was prepared with high radiochemical yields at room temperature, the same as for ^99m^Tc-HYNIC-annexin A5, which is a benchmark in the field of apoptosis imaging in nuclear medicine.

The main purpose of the drug design of ^99m^Tc-C_3_(BHam)_2_-annexin A5 is the lower accumulation and faster clearance of radioactivity from non-target tissues. It has been reported that persistent radioactivity localization in tissues after injection of [^99m^Tc](HYNIC-polypeptide)(tricine)_2_ could be attributed to the slow rate of elimination of the radiometabolite, [^99m^Tc](HYNIC-lysine)(tricine)_2_, from the lysosomes, due to the replacement of a tricine as a coligand with high molecular weight proteins [16]. In the biodistribution experiments in this study, ^99m^Tc-HYNIC-annexin A5 showed very high radioactivity in kidney; radioactivity in the organs for metabolism, such as liver and kidney, was retained for a long time (Table 1). In this case, the slow rate of elimination of radiometabolite from ^99m^Tc-HYNIC-annexin A5 might also affect the long retention of radioactivity in liver and kidney. On the other hand, when ^99m^Tc-C_3_(BHam)_2_-annexin A5 was administered to normal mice, the radioactivity in liver and kidney decreased gradually over time. As we expected, this result suggests that the radiometabolite of ^99m^Tc-C_3_(BHam)_2_-annexin A5 was not residual in the metabolic organs. However, after administration of ^99m^Tc-C_3_(BHam)_2_-annexin A5, the rate of blood clearance was slower and the accumulation of radioactivity in liver was higher than we expected. Introduction of the C_3_(BHam)_2_ ligand and linker might affect the biodistribution of annexin A5 although the bioactivity of ^99m^Tc-C_3_(BHam)_2_-annexin A5 remained. The slow blood clearance should hamper imaging soon after injection of the radiotracer. However, it could enhance the accumulation of the radiotracer at the lesion site. In a previous study, the introduction of polyethylene glycol (PEG) into ^111^In-labeled annexin A5 achieved increased uptake of and improved visualization with the radiotracer in solid tumors after chemotherapy [17]. Actually, ^99m^Tc-C_3_(BHam)_2_-annexin A5 showed higher accumulation in the tumor after treatment of 5-FU compared to ^99m^Tc-HYNIC-annexin A5 (Figure 3) although it may be not practival in this case because of the short half-life of ^99m^Tc.

To determine whether ^99m^Tc-C_3_(BHam)_2_-annexin A5 could detect early response after chemotherapy, 5-FU treatment for colon-26 tumor-bearing mice was performed. As a result, the treatment with 5-FU inhibited tumor proliferation in a dose-dependent manner (Figure 2). At the same time, ^99m^Tc-C_3_(BHam)_2_-annexin A5 accumulation in tumors 24 h after the first 5-FU treatment was also significantly increased in a dose-dependent manner compared to that of the untreated control group (Figure 3A). At the same time, TUNEL staining, which marks the degraded DNA in the cell, has been used as a marker for apoptosis [18]. The number of TUNEL-positive cells in the tumor sections of the 5-FU-treated groups was also greater than that of the untreated control group (Figure 4), and was significantly correlated with ^99m^Tc-C_3_(BHam)_2_-annexin A5 uptake or ^99m^Tc-HYNIC-annexin A5 uptake in tumor tissue (Figure 5). These results indicate that ^99m^Tc-C_3_(BHam)_2_-annexin A5 and ^99m^Tc-HYNIC-annexin A5 could predict therapeutic effects just after the start of 5-FU treatment before morphologic changes in tumor can be observed. Furthermore, our results are consistent with a previous report, which showed that tumor uptake of ^99m^Tc-HYNIC-annexin A5 increased significantly after a single dose of cyclophosphamide treatment (150 mg/kg, i.p.) in KDH-8 hepatoma tumor-bearing rats, and the increase was concordant with the number of TUNEL-positive cells in the tumor [19].

To investigate intratumoral distribution of ^99m^Tc-C_3_(BHam)_2_-annexin A5 and apoptotic cells, autoradiographic experiments and TUNEL staining were performed using serial frozen sections of tumor. Radioactivity and TUNEL-positive cells were heterogeneously distributed in the tumors, but the site of high accumulation of ^99m^Tc-C_3_(BHam)_2_-annexin A5 corresponded to the site of intense TUNEL-staining (Figure 6), which was a similar result to a previous report [17]. Moreover, the correlation between the intratumoral accumulations of radioactivity by autoradiography and the TUNEL-staining positive cells in corresponding sections was strongly positive and significant (Figure 7). These results also suggest that ^99m^Tc-C_3_(BHam)_2_-annexin A5 accumulates in lesions where apoptotic cells exist, that is to say, ^99m^Tc-C_3_(BHam)_2_-annexin A5 could be used to visualize apoptotic cells.

In conclusion, these findings suggest that ^99m^Tc-C_3_(BHam)_2_-annexin A5 may contribute to improved cell death imaging, namely, be useful in the rapid and efficient detection of apoptotic tumor response after chemotherapy.
